# Gamblers’ perceptions of responsibility for gambling harm: a critical qualitative inquiry

**DOI:** 10.1186/s12889-022-13109-9

**Published:** 2022-04-12

**Authors:** Sarah Marko, Samantha L. Thomas, Kim Robinson, Mike Daube

**Affiliations:** 1 Institute for Health Transformation, Faculty of Health, Deakin University, Melbourne, Australia; 2 School of Health and Social Development, Faculty of Health, Deakin University, Melbourne, Australia; 3 Faculty of Health Sciences, Curtin University, Perth, Australia

**Keywords:** Gambling harm, Public health, Personal responsibility, Campaigns, Qualitative, Tropes, Framing, Australia

## Abstract

**Background:**

Gambling has traditionally been conceptualised as an issue of addiction and personal responsibility. While there are now clear public health models that recognise that gambling harm is caused by a range of socio-cultural, environmental, commercial and political determinants, government and industry messages about gambling are still largely personal responsibility focused. Given the well-recognised issues associated with personal responsibility paradigms, this study sought to understand how gamblers themselves conceptualised responsibility for gambling harm.

**Methods:**

A qualitatively led online panel survey was conducted with 363 adult gamblers in New South Wales and Victoria, Australia. Participants were asked to respond to what they thought were the causes of gambling harm, and what could be done to prevent harm. A reflexive thematic analysis was conducted.

**Results:**

Six common tropes were constructed from gamblers’ responses: (1) Gambling in moderation; (2) Personal responsibility for rational behaviour; (3) Character flaws; (4) Personal responsibility to seek help; (5) More education is needed; and (6) Governments are responsible for action – but motivation and efficacy are questioned. Gamblers primarily understood gambling harm as being a matter of personal responsibility, and government responsibility was generally seen as limited to providing information to facilitate informed gambling choices.

**Conclusions:**

This study demonstrates that gamblers’ perceptions of gambling harm are similar to the personal responsibility framings and tropes present in industry and government messaging strategies. Refocusing public communication strategies away from ‘responsible gambling’ messaging, and towards evidence-based approaches, will be an important part of addressing the harms associated with gambling.

## Background

Gambling is a public health issue that has the potential to cause significant harm to individuals, their families and communities [1]. There are a range of harmful consequences that can result from gambling [2, 3], including family conflict [4], anxiety and other mental health disorders [5, 6], homelessness [7], suicidality [8], and intimate partner violence [9]. There is increasing evidence that harm may be experienced across a continuum of gambling behaviours [10]. For example, research conducted in 2018/19 in the Australian state of Victoria found that 70% of the harms reported by gamblers were experienced by people who were not classified as being high risk or ‘problem’ gamblers [11]. The study also reported that 6.1% of the adult population in Victoria were harmed by another person’s gambling [11]. This demonstrates that consideration of the harmful impacts of gambling should not be confined to those who experience problem or pathological gambling.

Gambling has traditionally been presented as an issue associated with personal responsibility and informed choice, with discourses from governments and the gambling industry focusing on the ‘responsible gambling’ paradigm of harm minimisation [12]. These discourses frame gambling as a recreational form of entertainment, and present gamblers as rational decision-makers who are able to make informed choices based on information regarding responsible gambling behaviours [13, 14]. These types of personal responsibility frameworks have been described as being *“overly simplistic”,* and ignoring the power differentials between individuals, governments, and the gambling industry [15]. While personal responsibility is of course an important part of gambling harm prevention, Orford [2017] argues that a dominant focus on responsible gambling paradigms creates a perception that gambling products are essentially unproblematic, and that any harms that may be associated with gambling are exceptional and can be minimised [16]. Such a focus not only places the emphasis on individuals who gamble, but also distracts from the role of the gambling industry in encouraging and promoting gambling, and the role of government in appropriately regulating the industry’s activities.

Researchers have also argued that unhealthy commodity industries use personal responsibility frameworks to minimise their role in the production of harm [17]. By framing individuals as informed consumers who are free to choose their health behaviours, personal responsibility paradigms also conflate the choice to engage in certain behaviours with the acceptance of responsibility for any harm that may be experienced [18]. This type of framing has contributed to the moralising of health behaviours by creating the perception that there are ‘good’ and ‘bad’ ways that individuals may consume or engage with products that may be harmful for their health [19]. From this perspective, the experience of harm signifies a moral failing of the decision-making agent [20], thus overlooking the range of factors that may contribute to the risks associated with gambling. Researchers have argued that personal responsibility framings may also contribute to the stigmatisation of individuals who are perceived to be at fault for their own negative experiences with gambling and other harmful products [21–25]. Researchers in gambling, and other areas of health, have clearly shown that when personal responsibility messages are internalised, they may result in stigma, stress and worry, lead to worse overall health outcomes, and create greater health disparities [20, 23, 24, 26, 27].

Researchers, politicians, peak bodies, and expert inquiries have called for broader conceptualisation of gambling harm and a public health response to harm prevention [1, 14, 28–32]. For example, in 1999 and again in 2010, the Australian Productivity Commission recommended a range of responses to gambling problems which moved away from personal responsibility strategies as the main harm minimisation response to gambling [33, 34]. These included strategies to reduce the risk and intensity of gambling products, and greater government regulation of gambling products and environments [35]. In New Zealand, a public health framework has been embedded in gambling legislation since the *Gambling Act 2003*, with a requirement for strategies to be “*focused on public health*” and to contain measures “*to promote public health by preventing and minimising the harm from gambling*” [36, 37]. More recently, three major reports in the United Kingdom have also concluded that gambling should be treated as a public health issue, moving away from responsible gambling paradigms towards strong regulatory environments needed to protect individuals, their families, and communities [38–40].

Despite evidence of community support for an increase in government regulation of gambling [41–43], public messaging strategies about gambling harm, including government and industry education campaigns, still largely focus on consumer choice and responsible gambling paradigms [12]. Recent government campaigns have also focused on individual responsibility strategies aimed at enabling gamblers to make informed choices; messages have included “*show gambling who’s boss*” by seeking help [44], “*become the type of man who controls the bet*” [45], and “*you’ve got the power*” to set time and money limits [46]. Alexius [2017] argues that the dominance of the individual responsibility discourse in gambling, and the lack of counter-framing of this discourse, may in fact contribute to the harm that agencies are trying to prevent:

If everyone in the gambler’s vicinity is taught that it is, in fact, the gambler’s own responsibility, and if this message is repeated everywhere the gambler turns in and around the market …. this becomes a powerful manifestation of an established responsibility order that is [sic] becomes difficult to escape and notions like ‘they must be right, there is no one to blame, it is all up to me’ will likely be internalized in many problem-gamblers [47].

While researchers have explored responsibility ‘tropes’ from the gambling industry and government [12, 47, 48], few studies have explored how gamblers themselves conceptualise responsibility for gambling harm. This is important in starting to understand how dominant framings about gambling risk and harm may influence how gamblers themselves conceptualise gambling. This study aimed to provide a starting point for such investigations. The study focused on three research questions:

1. How do gamblers describe the role of responsibility in relation to the causes of gambling harm?
2. Where do gamblers perceive the responsibility lies for the prevention of gambling harm?
3. What are the common ‘tropes’ in these descriptions of responsibility?

## Methods

### Approach

The data presented in this paper were part of a larger study exploring gamblers’ conceptualisations of gambling risk and harm. The study used a critical qualitative inquiry approach which applies a social justice lens to address power, inequality, and injustice to improve the social order [49, 50]. Green and Thorogood [2018] propose that critical researchers cannot and should not conduct value-free research [50]. We view gambling from a public health perspective, recognising that there are a broad range of individual, socio-cultural, environmental, commercial and political determinants that may contribute to gambling harm, and that this harm is not confined to those experiencing problem or pathological gambling behaviours. Deakin University Human Research Ethics Committee (HEAG-H 227_2020) gave ethical approval for the study.

### Why use an online qualitative survey?

Qualitatively focused online surveys are a relatively new method for qualitative data collection. They involve a series of self-administered open-ended questions [51]. There are a range of unique benefits and disadvantages in implementing online qualitative surveys.

First, the anonymity of online qualitative surveys may be a unique tool for reaching those who may otherwise be reluctant to participate in in-depth interviews [52]. This is particularly important in the area of gambling, in which experiences of ‘problem’ or ‘harmful’ gambling are highly stigmatised (arguably due to the dominant responsibility paradigm that underpins gambling harm minimisation efforts), particularly among some population sub-groups such as women, and individuals from culturally and linguistically diverse communities [53, 54]. The anonymity of online qualitative surveys may also help to address power dynamics between researchers and the participant, allowing increased opportunities for participants to challenge researcher assumptions or to challenge any perceived agendas [51, 55]. Second, while in-depth interview studies focus on small sub-groups of individuals and look for depth of meaning and experience, online surveys aim to gather smaller ‘chunks’ of textual data from a broader and more diverse population [51, 56]. Third, online qualitative surveys provide a fast method of collecting qualitative data [55]. Data collection, including piloting and replacing low-quality responses, took a total of twenty-three days over a two-month period. The downside of this method is that some of the aspects of qualitative research that we value are largely lost, such as the ability to prompt participants, reflect and explore new areas of thought or interest as data are gathered, and the ability to co-create data with participants.

### Sample and recruitment

Purposive sampling was used to recruit regular gamblers which we defined as gambling at least once in a typical month using Electronic Gambling Machines (EGMs, pokies, slot machines) or sports betting. The researchers aimed to recruit adults over the age of 18 years who lived in New South Wales or Victoria (Australia’s two most populous states). Individuals were recruited using the online software company Qualtrics. Qualtrics accessed potential participants through a range of panel-based databases comprised of individuals who had signed up to participate in online surveys. Individuals who met the demographic criteria were provided a link to information about the survey. If they wanted more information, they were provided a link to the Plain Language Statement and completed three eligibility questions to ensure that they met the study inclusion criteria. Consent was assumed by participants’ decision to begin and submit the survey. Soft quotas for age (18–29, 30–45, 46–60 and 60 + years) and gender (even split between males and females) were employed to ensure some diversity in the sample. As there are no specific guidelines for the ideal sample size when using qualitative surveys, we followed recommendations from Malterud et al. [2016] to ensure that the sample would provide enough ‘information power’ to be able to address the research aim and questions [57]. We aimed for a sample size of 400 participants because our aim had a broad focus, we wanted a sample that was diverse in age and experience, and the types of questions elicited shorter rather than longer textual-responses [55, 57]. Upon completion of the survey, participants received points from the online panel survey company from which they were recruited which could be traded for a range of products.

### Data collection

In relation to the data presented in this study, participants were asked a number of questions relating to their socio-demographic (age and birthplace) and gambling characteristics (the type and number of gambling products used in a typical month). The data interpreted for this paper were based on responses to three open-text questions relating to the causes of gambling harm, strategies to prevent gambling harm, and if the participants had anything else to write about gambling harm. We did not ask specifically about responsibility to avoid leading participants into focusing on this issue. Rather we were interested in their top-of-mind responses to these issues.

Piloting was used to assess the comprehension and clarity of questions, and to determine whether follow-up questions needed to be introduced to help guide participants in providing detailed responses [55, 58]. For example, we considered whether it was clear participants understood the questions, if their responses matched the intended meaning of the questions, and whether there were a large number of non-responses which may have indicated the question was too difficult. Responses from all participants were checked for quality of response, with 31 participants who provided inconsistent or unreliable data (for example, those who provided nonsensical responses to qualitative questions) removed from the data and replaced with new participants. While analysing the data, an additional 64 participants were removed due to discrepancies within their responses. For example, despite initially agreeing they met the inclusion criteria, when asked about their gambling behaviour within the survey they stated they never or rarely gambled using EGMs or sports betting.

### Data interpretation

Braun and Clarke’s approach to reflexive thematic analysis was used to interpret the data. Data interpretation involved moving between six phases of analysis [59–61]. This included reading and re-reading the data to become familiar with it while noting thoughts about responsibility. Author 1 led the coding process with a specific focus on how participants described responsibility in relation to the development and prevention of gambling harm. Initial coding focused primarily on semantic codes, representing the surface-level meaning of the text. However, where possible, and where participants provided more detailed responses, latent codes were also included to reflect the underlying assumptions. The codes were collated, refined, and grouped according to patterns of related meaning – or tropes. Tropes refer to the recurrent motifs that reflect a limited way in which a story is presented and which signal “*a lexicon, a way of talking and thinking about issues*” [62]. We focused on tropes because they provided a unit of analysis which could capture the common ideas in how gamblers spoke about the causes of gambling harm. Quotes have been included to illustrate the tropes. These have primarily been left as they were written by the participants; however, some misspellings and grammatical errors have been corrected for clarity.

**Fig. 1:**
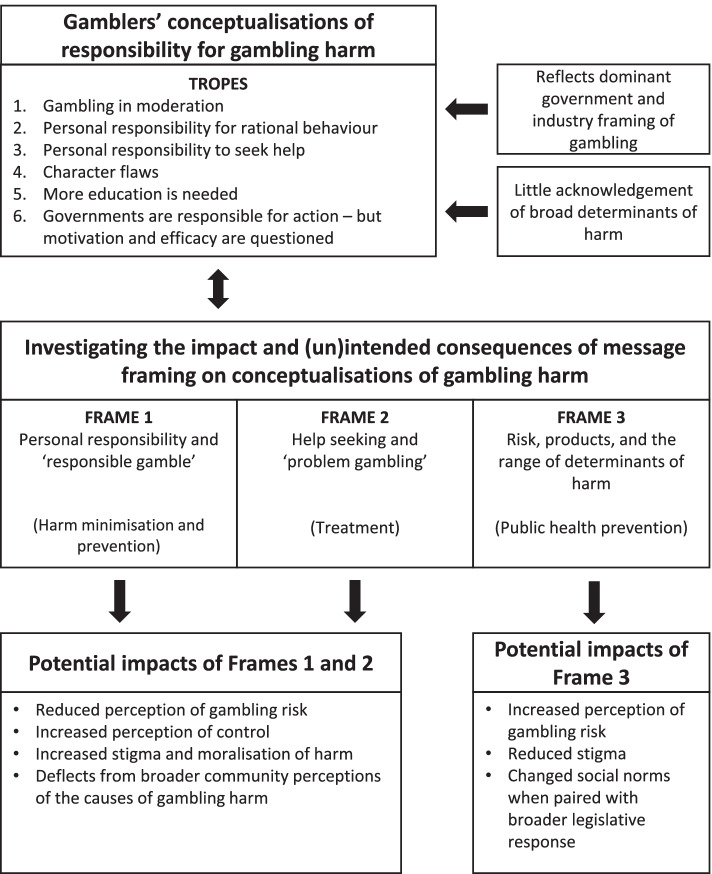
A proposed model of the impact of responsibility framing of gambling harm

To ensure reflexivity and the rigour of the analysis, the authors met regularly to discuss the coding process, the themes that were constructed from the data, and how these could be explained according to existing literature. We developed a theoretical model (Fig. 1) from the data to illustrate the key themes and associated concepts in light of the research questions and literature.

## Results

### Socio-demographic and gambling characteristics

Table 1 provides detail about the socio-demographic and gambling characteristics of the participants. A total of *n* = 363 gamblers participated in the survey following the removal of ineligible participants. Participants had an average age of 45.22 years (range 18–87 years, SD 17.83). Over half of participants lived in New South Wales (*n* = 217, 59.8%), and the vast majority were born in Australia (*n* = 290, 79.9%). EGMs were used by most participants in a typical month (*n* = 268; 73.8%), followed by lotteries (*n* = 231; 63.6%), and sports betting (*n* = 185; 51.0%). Almost two thirds of participants used three or more gambling products in a month (*n* = 218; 60.1%).

**Table 1:** Participant socio-demographic and gambling characteristics (*n* = 363)

	**Frequency**	**Percentage**^**a**^
**Gender**		
Male	185	51.0%
Female	178	49.0%
**Geographic location**		
New South Wales	217	59.8%
Victoria	146	40.2%
**Age**		
18–29	99	27.3%
30–45	89	24.5%
46–60	90	24.8%
60 +	85	23.4%
**Country of birth**		
Australia	290	79.9%
United Kingdom	16	4.4%
India	14	3.9%
China	10	2.8%
Other	33	9.1%
**Education**		
Secondary school education	114	31.4%
Trades-based education	105	28.9%
Tertiary education	144	39.7%
**Employment status**		
Working full-time	165	45.5%
Working part-time / casually	67	18.5%
Retired	60	16.5%
Homemaker	22	6.1%
Unemployed but looking for work	20	5.5%
Full-time student	14	3.9%
Other	15	4.1%
**Income per week**		
Over $3000	39	10.7%
$2500—$2999	39	10.7%
$2000—$2499	51	14.0%
$1500—$1999	65	17.9%
$1000—$1499	82	22.6%
$500—$999	59	16.3%
$499 or less	27	7.4%
No income	1	0.3%
**Gambling products used in a typical month**^**b**^		
Electronic gambling machines	268	73.8%
Lotteries	231	63.6%
Sports betting	185	51.0%
Scratch cards / scratchies	166	45.6%
Horse betting	128	35.2%
Casino games	70	19.2%
**Number of gambling products used in a typical month**		
1	54	14.9%
2	91	25.1%
3	120	33.1%
4 +	98	27.0%

^a^Totals may not add up to 100% due to rounding

^b^Participants could select multiple responses

Six tropes were developed from the data. These tropes reflected common ideas described by participants that demonstrated the interaction between the gambler as being responsible for causing harm, and the shared responsibility between the gambler and the government to prevent gambling harm from occurring: (1) Gambling in moderation, (2) Personal responsibility for rational behaviour, (3) Character flaws, (4) Personal responsibility to seek help, (5) More education is needed, and (6) governments are responsible for action – but motivation and efficacy are questioned. Importantly, the tropes were not mutually exclusive and there is some overlap. For example, making informed decisions and the government’s responsibility to facilitate this also assumes that gamblers are rational decision-makers.

### Trope one: gambling in moderation

Many participants wrote about how gamblers were responsible for moderating how they spent money on gambling. There was a focus in the written responses on financial responsibility, in the sense that gamblers needed to ensure they did not spend ‘*too much*’ or an ‘*excessive*’ amount of money. Gamblers were described as being responsible for identifying a logical point to stop gambling based on how much they could ‘*afford to lose*’. Some participants suggested strategies that individuals could implement to ensure they gambled in moderation, such as setting money aside specifically for gambling, only gambling on certain days, and betting small amounts. Several participants reflected the responsible gambling paradigm in noting that choosing to gamble was a relatively straightforward personal choice for individuals.

“*If you cannot afford it don’t do it…*” – 66-year-old male

For a few participants, an individual’s gambling was harmful when the financial losses impacted their ability to comply with social norms relating to financial responsibility. Harm was perceived to occur when gamblers could not meet their financial obligations because money was redirected to gambling from “*essential*” payments such as “*bills*”, “*mortgage*”, “*rent*” or “*food*”. Some participants distanced their own gambling behaviours from the behaviours of those who had experienced harm. For example, some participants described their own gambling as being harmless because they gambled within the boundaries of moderation.

“*My release is my pokies, which I enjoy playing, however, I know when it’s time to go, without being told by my husband and I also know within my mind what is fair in my mind to put in. We each have our pleasures in life, his is cars, give and take on both parts without going over the boundary, works well for us.*” – 65-year-old female

### Trope two: personal responsibility for rational behaviour

The majority of participants perceived gambling to be a matter of personal responsibility. They commented that gamblers should make rational decisions about participating in gambling. Many participants stated that preventing harm from gambling was relatively simple if gamblers controlled their behaviour or, as one participant stated, made “*better choices*”. Participants’ comments reflected many of the slogans present in Australian responsible gambling messages. For example, participants stated that when individuals were gambling they should “*know when to stop*”, “*stick to the rules*”, “*set limits*”, and “*gamble responsibly*”.

“*People can gamble responsibly as long as they stick to the few rules you set.*” – 41-year-old female

Some participants wrote that rational decision making and personal responsibility applied even within the context of compulsive gambling behaviours. While some recognised that compulsive gambling behaviour was beyond the control of the individual, they also stated that it remained the gambler’s responsibility to intervene. For example, some participants wrote that the gambler needed to implement responsible gambling strategies to counter the addictive behaviour, and if they were unable to control the behaviour they needed to “*stop gambling*” or remove the temptation by “*staying away*” from gambling venues.

“*Not going out as much to places where there are temptations and have a really good support system to help you.*” – 18-year-old female

Others focused on the individual avoiding gambling by replacing it with something more “*productive*” and distracting themselves by “*keeping busy*”. This included spending time with friends and family, adopting a new hobby, or finding new ways to use the money instead.

“*Get a future goal and start saving money for it, join social activities, travel, spend time with family.*” – 25-year-old male

### Trope three: character flaws

Some participants commented that gamblers who made irresponsible decisions about gambling had a range of character flaws, including that they were “*greedy*”, were “*lacking willpower*”, or were “*weak minded*”. For example, the following participant felt that excessive or irresponsible gambling was due to a lack of intelligence, or stupidity.

“*You cannot stop dumb people from doing dumb things.*” – 67-year-old male

Some participants demonstrated a lack of empathy towards those who experienced harm because they had not experienced it themselves. When participants considered it was easy to control their gambling behaviours, some assumed others experienced harm because there was something wrong with them. For example, the following participant described how people “*inflict*” harm on themselves because they were perceived to gamble for selfish reasons and believed they could win. By comparison, he stated he had not experienced harm because he gambled for social reasons and expected to lose money.

“[Gambling harm is caused by] *greediness. People thinking that they can win by gambling which is only a rare occasion. I play the pokies to be with friends and enjoy ourselves. We all know that there is a 90% chance of losing what you set aside to gamble with…It is all self-inflicted and you are the only one that can prevent it. No use blaming anyone nor anything else…I have never ever had to do without anything as a result of gambling.*” – 76-year-old male

### Trope four: personal responsibility to seek help

The most commonly expressed opinion was that gambling harm was the result of a gambling addiction or a compulsive behaviour. However, gambling addiction was seen as being a treatable condition as long as the gambler sought help. Most participants used only a few words to describe addictive behaviours – “*addiction*”*,* “*obsession*”, “*compulsion*”, “*disease*”. These straightforward and short responses suggested that some participants viewed the cause of gambling harm as being unambiguous, straightforward, and largely attributable to a loss or lack of control over behaviours. Most participants recognised that individuals had very limited control over their addictive gambling behaviours. However, many participants also stated that individuals were personally responsible for taking proactive measures to seek help for something that they perceived could be treated. For example:

“*I think it’s an addiction, it’s treatable however the person needs to seek help.*” – 56-year-old male

This also included gamblers recognising and admitting they had a ‘*problem*’. For example, some stated that gamblers had a responsibility to ‘*speak up*’ or ‘*get help*’ to prevent harm.

“*…the individual taking the first steps to admitting their addiction and getting help and assistance with programs that could potential change your aspects or gambling.*” – 21-year-old female

Participants identified a range of services that individuals could contact if they experienced problems, including Gamblers Helpline, Gamblers Anonymous, psychologists, counsellors, and rehabilitation services.

### Trope five: more education is needed

Some participants commented that gamblers would be better able to make informed gambling choices if they were provided with more information about the realities of gambling. Therefore, the government and industry were described as having a responsibility to “*raise awareness*” and provide “*education*”. Participants identified two key areas for potential education campaigns to improve informed choice. The first area related to the potential harms associated with gambling. It was suggested that individuals would be able to make informed choices if they better understood the “*effects*”, “*harm*” and “*dangers*” associated with gambling and gambling addiction. Schools were identified as being a potential setting for education to prevent young people from engaging in gambling. For example, one younger female participant stated that she did not learn about the negative effects of gambling until completing the training required to work in a gambling venue, concluding that the risks of gambling should be taught earlier, and targeted towards particular population subgroups.

“*It should be talked about more in high school, especially to males. I learned a lot about the effects when I did my Responsible Service of Gaming but I think this should be taught in high school.*” – 23-year-old female

The second area for education related to correcting misunderstandings about the potential outcomes of gambling. Participants described how some gamblers believed they could “*chase*” or “*win back*” money they had already lost, while others described the idea of “*winning big*” as enticing people to continue gambling. In these circumstances, the decision to gamble was based on the misinformed belief they could influence the outcome of the gamble or that winning was likely. Thus, individuals who viewed gambling as “*easy money*” or a way to “*improve their fortunes*” and “*live a life of luxury, debt free*” were perceived to be at risk of harm. Therefore, there was an understanding that individuals would make better choices and avoid gambling harm if they expected to lose the money they gambled with. For example, this participant believed people should be able to enjoy the experience of gambling but accept that only the gambling operators will benefit.

“*Gambling in order to win money is very dangerous, better to expect to lose the money…Gambling is something that can be enjoyed by many people, but ultimately at the end of the day the bookie always wins because it is a business…*” – 20-year-old male

### Trope six: governments are responsible for action – but motivation and efficacy are questioned

A few participants stated that the government had a responsibility to better regulate gambling environments and products. Some of these participants wrote that gambling products had become too easily accessible which was contributing to the normalisation of gambling. In their view, this was because there has been an increase in the types of gambling products in Australia, EGMs were increasingly accessible in community settings (“*every pub has them*”), and venues were allowed to operate for longer periods each day (“*at all hours of the day*”). One participant expressed frustration with the government and compared the current gambling environment to when it was more restricted in the past.

“*Shame on governments to permit gambling operators to ever increasing ways of gambling. Not so long ago racing occurred Wed, Thurs and Saturdays. Now 24/7.* [There use to be] *no legal sports betting. Now again 24/7. There were no pokies. Now up to 21 hrs daily. There was no casinos (only 1 in Tassie). Now* [casinos are] *each states’ biggest employers ... could go on and on but do not have a week to spare.*” – 56-year-old male

Other participants responded that the government also had a responsibility to better regulate gambling products to ensure they were safer for those who chose to use them. These participants stated that “*limits*” or “*restrictions*” could be introduced at the population level, for example through a system to limit the amount of time and money that could be spent on gambling products. Rather than requiring gamblers to implement their own limits or targeting specific at-risk gamblers, these participants described mechanisms which would require everyone who chooses to use gambling products to adhere to pre-determined limits. One participant suggested that limits should be set based on an individual’s income “*like credit cards*” to ensure affordability while others suggested a maximum “*bet limit*”. Most of these participants focused on limiting EGMs and venue-based gambling products. Potential mechanisms to enforce limits included a registration or identification system which would lock individuals from accessing products for a period of time if they reached a pre-determined limit. However, one participant explained that this would need to be implemented across all venues in order to be effective.

“*Machines register each player with a card and all patrons have a daily limit on gambling, all venues would have to be connected for this to work.*” – 49-year-old female

While some participants supported better regulation of gambling or the closure of venues, a few were critical of government interventions. These criticisms primarily came from a perspective that gambling was a “*personal*” decision and governments should not limit individual’s agency as the decision-maker. One concern was that interventions would limit their own ability to gamble. As those who experienced harm were perceived to represent a small portion of gamblers, there was a perception that the majority of gamblers who gambled safely and responsibly should not be impacted because of the behaviour of others. For example, one participant expressed concern that people who do not experience addiction may be unfairly targeted, and interventions should focus on the reason individuals develop addiction.

“*Most people gamble and have fun. A lot don't become problem gamblers so I think you can't just come down on all those who do gamble. There's usually other reasons associated with gambling that cause addiction. E.g., loneliness. All factors should be considered to one’s addiction not just the fact they gamble.*” – 49-year-old male

A few participants also questioned the government’s ability to effectively regulate gambling and expressed a mistrust regarding their motivation to intervene. One participant believed governments viewed gambling taxation as “*easy money*” and “*preyed on vulnerable people* [by] *encouraging gambling*”. Two participants supported increased regulation but suggested that government reliance on gambling revenue meant that reform was unlikely and that governments may be motivated by their own interests. One participant compared gambling to other unhealthy commodities the government also taxes and implied that the government was conspiring with industry to ensure they received their “*share*” of the revenue.

“*…they need to close the venues that have gambling, but that won't happen as the Govt want their share of the takings the same as for cigs and beer.*” – 55-year-old female

## Discussion

This study sought to understand how gamblers conceptualise gambling harm. By examining the range of tropes that appeared in gamblers’ textual responses to questions about the causes and prevention of gambling harm, the study provides important preliminary information about how gamblers conceptualise the responsibility for gambling harm. Tropes are central to public and commercial messaging strategies [63]. By examining the tropes in participants’ responses, this study further provides new information about how gamblers ascribe meaning to gambling harm, and suggests approaches to guide the development of future public health research and strategies. Figure 1 provides a conceptual model of the tropes that were constructed from the data and suggests areas for future research to investigate how current dominant frames about gambling may impact upon gamblers’ conceptualisations of harm. This has important implications for both messaging from health authorities and the regulation of gambling industry promotional activities and materials.

The findings from this study raise a number of points for discussion about the role of personal responsibility paradigms in gambling.

First is gamblers’ top-of-mind responses about the role of personal responsibility in gambling harm. Participants in this study overwhelmingly stated that individuals were to some degree personally or morally responsible for both the causes and outcomes of gambling harm. This included their own failure to gamble in moderation, their inability or unwillingness to make rational gambling decisions, their character flaws, and their failure to seek help as soon as their gambling became a problem. These responses overwhelmingly reflected messages that have been identified in the literature as being prominent in industry and government discourses about gambling. Research has demonstrated that the gambling industry, governments, and associated industry-funded bodies use a range of framing strategies to reinforce that gambling is similar to other legal entertainment products that individuals freely choose to engage in, that problem gambling is a relatively small problem that has not increased over time, and that gamblers have the personal responsibility to monitor their gambling, maintain self-control and seek help [64]. Miller and colleagues’ [2016] detailed investigation of industry and government discourses about gambling found that messages were largely framed around responsibility, individual control, and self-monitoring of behaviours [12]. In particular, industry and government messages included a range of expectations around rational behaviour, such as that gamblers are required to “*exercise constant vigilance over their behaviour, to identify signs that their gambling may no longer be ‘responsible’*” and to seek help if their behaviour no longer meets expected standards of responsibility [12].

The findings in this present study are important because they demonstrate that gamblers have almost identical tropes as those seen in gambling industry and government discourses about gambling harm. Alexius [2017] states that with a lack of any notable counter-framing to personal responsibility messages, it is likely that these messages about gambling have been largely internalised by gamblers [47]. While personal responsibility is of course an important part of many public health issues, an overwhelming emphasis on personal responsibility as the primary driver of behaviours may arguably cause more harm than good [20, 26], particularly if these messages internalise, or create blame and shame in the very people they are trying to help [27]. In this context, there appears to be no reasonable rationale for an overwhelming focus on personal responsibility framings in public education about gambling. Researchers have noted that there is no clear evidence or relevant independent research that supports such approaches, with government interventions often bearing little resemblance to best practice evidence for preventing gambling harm [13].

Other areas of public health such as tobacco and alcohol have demonstrated the benefits of evidence-based and independent public education programs [65, 66]. Tobacco research has demonstrated that not only is the message framing important, but also the source of the message, with research clearly demonstrating that prevention campaigns from the tobacco industry were less effective than anti-smoking campaigns developed by public health organisations (for a review see [67]). Following the precedent set by the WHO Framework Convention for Tobacco Control [68], which has been ratified by 168 countries, and recommendations from the WHO that specifically note the “*fundamental and irreconcilable conflict between the tobacco industry’s interests and public health policy interests*” [69], we recommend that governments should exclude the gambling industry from any involvement in the formulation and implementation of public health policies or education programs, and avoid aligning their messaging with that of the industry.

It is now well recognised in the gambling literature, and many government policy statements, that gambling is a complex public health problem [1, 37, 70, 71]. New policy and practice paradigms recognise the need for public health models which recognise the complex range of determinants that may contribute to harm [13, 14]. However, these evidence-based shifts in how gambling harm has been conceptualised may not have translated to how gamblers as a group think about harm. Rather there are still significant moralising stereotypes amongst gamblers about the ‘irresponsible’ consumer, and clear divides between how gamblers conceptualise their own responsible behaviours and the behaviours of others. These findings are similar to a study which found gamblers also describe gambling problems as being a matter of irresponsible behaviour and moral failings [27]. In the present study, these were particularly strong in relation to expectations about how gamblers should manage money. We would argue that these entrenched tropes, and the embedding of ideologies about responsibility and control (particularly in relation to the management of money), provide significant challenges in implementing public health responses to gambling harm. Personal responsibility paradigms contribute to these challenges because they determine which health behaviours are considered socially and morally acceptable and which should be sanctioned [72]. Reflecting research findings regarding the moralisation of alcohol use and misuse [73], this study has noted that some gamblers consider themselves morally superior to those who experience harm because they believe their gambling matches the societal expectations of responsibility. These expectations are reinforced through messaging about personal responsibility.

Another point for discussion relates to governments’ responsibility to protect the health and wellbeing of the community. For example, the *Victorian Public Health and Wellbeing Act 2008* recognises that the State has “*a significant role in promoting and protecting the public health and wellbeing of persons in Victoria…promoting conditions in which persons can be healthy*” [74]. This study found that gamblers largely perceived that the government’s responsibility was limited to providing education to help consumers make informed choices. While acknowledging that providing honest risk information is important, Hodgins argues that personal choice is complex and, when considering the successful reduction of tobacco use, that changing consumer behaviour requires government regulation of the industry [15]. Legislative requirements to promote conditions in which persons can be healthy also provide important direction for responding to the challenges and controversies relating to the use of personal responsibility paradigms in gambling. This includes addressing the polarising and largely negative outcomes of the framing of gambling as an issue associated with personal responsibility. One way of addressing this is to use legislation to reduce the potential for individuals to engage with gambling products in a risky or harmful way, such as restricting access to high intensity gambling products [75], and reducing maximum bet sizes and venue opening hours [33, 34]. This may also help to remove some of the moralising and stigmatising discourses that were identified in the current study around individuals who are perceived to be unable to engage with products and monitor their own behaviours in a responsible way. It would also be consistent with public health approaches which recognise that gambling products and marketing are the key vectors of harm [76]. This study found that many individuals had a level of perceived competency and confidence with their ability to gamble ‘responsibly’. However, numerous studies show that gambling environments, products, and marketing make it difficult for some individuals to meet the standards and expectations of responsibility that have been demanded by industry and government [48, 77–79]. Therefore, given the dominance of personal responsibility in gamblers’ responses, we would argue that governments have a legislated obligation to move beyond and away from the promotion of responsible gambling paradigms to address the broader determinants of harm. As has been clearly demonstrated in other areas of public health, strong curbs on marketing, the regulation of products (including the components and ingredients), and the right to honest information about the products create conditions which support individuals to engage in ‘responsible’ ways without compromising individual freedom and choice [80–82] are all important components of a comprehensive public health approach. These universal protections may also help to prevent the negative health outcomes and stigma that may eventuate from not being able to be ‘responsible’ with gambling [24], and money [26].

The tropes identified in this study provide further evidence for the need to refocus public communication away from unrealistic and stigmatising expectations of individual behaviour. This will help to support a broader legislative response, thus changing the social norms relating to gambling rather than focusing almost exclusively on the individual’s role in behaviour change. This type of refocusing has been effective in the tobacco arena in which there has been a substantial shift in tobacco control activity to exposing the activities and approaches of the industry – not just as part of advocacy but as a strong argument to cease or never start smoking [82–84]. While this study shows that some gamblers are critical of government involvement in what they consider are personal decisions, Moore and colleagues [2015] have described how government interventions could be reframed as providing freedom from the domination of industry by restricting exploitative industry behaviour rather than reducing personal freedoms [85]. Reframing the issue further towards irresponsible industry behaviour rather than solely focusing on gamblers’ responsible consumption behaviours could minimise the impact of those who argue that public health action is an inappropriate infringement on personal liberty and choice.

There are limitations associated with this study. While the sample was relatively large for a qualitative online survey [86], the findings cannot be generalised to all gamblers. The study was limited to those who are fluent in English and was also limited to two Australian states with distinctive gambling environments. The perspectives of people from culturally and linguistically diverse backgrounds and alternative gambling environments are important in developing a more comprehensive understanding of how different types of gamblers and their social contexts may influence conceptualisation of gambling harm.

## Conclusion

This study demonstrates how gamblers conceptualise and internalise gambling harm as an issue largely related to personal responsibility. The personal responsibility paradigm has a long tradition and has been the primary focus for public education about gambling from both the gambling industry and governments. The continued dominance of personal responsibility paradigms in gambling suggests that these types of messages may exacerbate negative health outcomes. There are clear benefits for both industry and governments in placing the burden squarely on the shoulders of individual gamblers because it conveniently releases them from any significant responsibility. The participants in this study provided evidence of the influence of personal responsibility messages via the tropes discussed and promulgated by industry and government. Refocusing public communication strategies away from ‘responsible gambling’ messaging, and towards research-based approaches will be an important part of addressing the harms associated with gambling.

## Data Availability

The dataset analysed in the current study is not publicly available, or available on reasonable request from the corresponding author because participants explicitly consented to only have their data shared with the immediate research team.

## Abbreviations

EGM: Electronic Gambling Machines
